# Supplementary material for: Resolving the full spectrum of human genome variation using Linked-Reads
Source: Genome Res. 2019 Apr;29(4):635–45. doi: 10.1101/gr.234443.118 (PMC6442396; doi:10.1101/gr.234443.118)
Supplement: Supplemental Material [file supp_gr.234443.118_Supplemental_longranger-2.2.zip › longranger-2.2.tar.bz2!/lr2.2-public/lib/stan/cmdstan/stan/doc/api/html/agrad_2rev_2functions_2gamma__q_8hpp_source.html]

Stan: src/stan/agrad/rev/functions/gamma\_q.hpp Source File


|  |  |
| --- | --- |
|  | Stan  2.5.0  probability, sampling & optimization |


- Main Page
- Related Pages
- Namespaces
- Classes
- Files

- File List
- File Members

All Classes Namespaces Files Functions Variables Typedefs Enumerations Enumerator Friends Macros Pages

- src
- stan
- agrad
- rev
- functions

gamma\_q.hpp

Go to the documentation of this file.

1 #ifndef STAN\_\_AGRAD\_\_REV\_\_FUNCTIONS\_\_GAMMA\_Q\_HPP

2 #define STAN\_\_AGRAD\_\_REV\_\_FUNCTIONS\_\_GAMMA\_Q\_HPP

3

4 #include <valarray>

5 #include <stan/agrad/rev/var.hpp>

6 #include <stan/agrad/rev/internal/vv\_vari.hpp>

7 #include <stan/agrad/rev/internal/dv\_vari.hpp>

8 #include <stan/agrad/rev/internal/vd\_vari.hpp>

9 #include <stan/math/functions/gamma\_q.hpp>

10 #include <stan/prob/internal\_math.hpp>

11 #include <boost/math/special\_functions/gamma.hpp>

12 #include <boost/math/special\_functions/digamma.hpp>

13

14 namespace stan {

15  namespace agrad {

16

17  namespace {

18  class gamma\_q\_vv\_vari : public op\_vv\_vari {

19  public:

20  gamma\_q\_vv\_vari(vari\* avi, vari\* bvi) :

21  op\_vv\_vari(stan::math::gamma\_q(avi->val\_,bvi->val\_),

22  avi,bvi) {

23  }

24  void chain() {

25  avi\_->adj\_ += adj\_

26  \* stan::math::gradRegIncGamma(avi\_->val\_, bvi\_->val\_,

27  boost::math::tgamma(avi\_->val\_),

28  boost::math::digamma(avi\_->val\_));

29  bvi\_->adj\_ -= adj\_

30  \* boost::math::gamma\_p\_derivative(avi\_->val\_, bvi\_->val\_);

31  }

32  };

33

34  class gamma\_q\_vd\_vari : public op\_vd\_vari {

35  public:

36  gamma\_q\_vd\_vari(vari\* avi, double b) :

37  op\_vd\_vari(stan::math::gamma\_q(avi->val\_,b),

38  avi,b) {

39  }

40  void chain() {

41  avi\_->adj\_ += adj\_

42  \* stan::math::gradRegIncGamma(avi\_->val\_, bd\_,

43  boost::math::tgamma(avi\_->val\_),

44  boost::math::digamma(avi\_->val\_));

45  }

46  };

47

48  class gamma\_q\_dv\_vari : public op\_dv\_vari {

49  public:

50  gamma\_q\_dv\_vari(double a, vari\* bvi) :

51  op\_dv\_vari(stan::math::gamma\_q(a,bvi->val\_),

52  a,bvi) {

53  }

54  void chain() {

55  bvi\_->adj\_ -= adj\_

56  \* boost::math::gamma\_p\_derivative(ad\_, bvi\_->val\_);

57  }

58  };

59  }

60

61  inline var gamma\_q(const stan::agrad::var& a,

62  const stan::agrad::var& b) {

63  return var(new gamma\_q\_vv\_vari(a.vi\_,b.vi\_));

64  }

65

66  inline var gamma\_q(const stan::agrad::var& a,

67  const double& b) {

68  return var(new gamma\_q\_vd\_vari(a.vi\_,b));

69  }

70

71  inline var gamma\_q(const double& a,

72  const stan::agrad::var& b) {

73  return var(new gamma\_q\_dv\_vari(a,b.vi\_));

74  }

75

76  }

77 }

78 #endif

dv\_vari.hpp

gamma\_q.hpp

stan::agrad::tgamma

fvar< T > tgamma(const fvar< T > &x)

**Definition:** tgamma.hpp:15

vd\_vari.hpp

stan::agrad::var::vi\_

vari \* vi\_

Pointer to the implementation of this variable.

**Definition:** var.hpp:40

stan::agrad::gamma\_q

fvar< T > gamma\_q(const fvar< T > &x1, const fvar< T > &x2)

**Definition:** gamma\_q.hpp:15

stan::math::gradRegIncGamma

double gradRegIncGamma(double a, double z, double g, double dig, double precision=1e-6)

**Definition:** internal\_math.hpp:181

var.hpp

internal\_math.hpp

stan::agrad::var

Independent (input) and dependent (output) variables for gradients.

**Definition:** var.hpp:27

stan::agrad::digamma

fvar< T > digamma(const fvar< T > &x)

**Definition:** digamma.hpp:16

vv\_vari.hpp


---

[ Stan Home Page ]

*© 2011–2014,
Stan Development Team.*
